# Perception and implementation gaps in active teaching and learning: a mixed-methods study of students and tutors in a medical degree program

**DOI:** 10.3389/fmed.2026.1796205

**Published:** 2026-05-21

**Authors:** Ibrahim Al Nabhani, Srinivasa Rao Sirasanagandla, Adhari Al Zaabi, Hussein Sakr, Halima Al Balushi, Mohammed Al Saidi, Meher Rizvi, Raya Al Maskari, Hajar Al Rajaibi

**Affiliations:** 1 Department of Hematology, College of Medicine and Health Sciences, Sultan Qaboos University (SQU), Muscat, Oman; 2 National Hematology and Bone Marrow Transplant Center, University Medical City, Musct, Oman; 3 Department of Human and Clinical Anatomy, College of Medicine and Health Sciences, Sultan Qaboos University (SQU), Muscat, Oman; 4 Department of Physiology, College of Medicine and Health Sciences, Sultan Qaboos University (SQU), Muscat, Oman; 5 College of Medicine and Health Sciences, Sultan Qaboos University (SQU), Muscat, Oman; 6 Department of Microbiology and Immunology, College of Medicine and Health Sciences, Sultan Qaboos University (SQU), Muscat, Oman; 7 Department of Pharmacology and Clinical Pharmacy, College of Medicine and Health Sciences, Sultan Qaboos University (SQU), Muscat, Oman

**Keywords:** active learning, faculty development, medical education, mixed-methods research, student engagement, tutorial-based learning

## Abstract

**Background:**

Active Teaching and Learning methods (ATLs) are increasingly incorporated into undergraduate medical curricula. However, discrepancies often exist between educators’ intentions and students’ experiences. This study explored the perceptions of medical students and tutors regarding active learning within tutorial-based sessions at a Middle Eastern medical school.

**Methods:**

A cross-sectional mixed-methods study was conducted at a Middle Eastern medical school. Phase II medical students and tutorial facilitators completed parallel perception surveys assessing engagement, satisfaction, and use of active learning strategies. Quantitative data were analyzed descriptively. Four student focus groups were conducted, and qualitative data were analyzed using inductive thematic analysis.

**Results:**

Fifty-two students and twenty-nine tutors participated. While most tutors reported frequent use of active learning strategies and high satisfaction with tutorial delivery, students reported limited engagement and low satisfaction, with only 9.6% rating tutorials positively. Qualitative analysis identified three key themes: tutor-dominated sessions with limited interactivity, misalignment between tutorial activities and learning objectives, and structural barriers including large group size and inadequate resources. Tutors acknowledged limited training and preparation time as additional challenges.

**Conclusion:**

Substantial perception gaps exist between tutors and students regarding active learning implementation. Although tutors report frequent use of active strategies, students often experience tutorials as passive and minimally interactive. Addressing these gaps requires structured faculty development in facilitation skills, reduction of tutorial group size, clearer alignment between tutorial cases and stated learning objectives, and systematic monitoring of implementation fidelity.

## Introduction

Active teaching and learning methods (ATLs) are widely promoted in undergraduate medical education because of their association with improved learner engagement, knowledge retention, and higher-order cognitive processing compared with traditional lecture-based teaching. A large meta-analysis by Freeman et al. (1) demonstrated that active learning significantly improves student performance and reduces failure rates in STEM disciplines. In medical education specifically, problem-based and case-based approaches have been shown to promote deeper understanding, integration of knowledge, and clinical reasoning skills (2, 3). As a result, many medical curricula worldwide have transitioned toward integrated, small-group, and case-based instructional models that emphasize collaborative learning and student participation (4, 5). These approaches are grounded in constructivist learning theory, which emphasizes knowledge construction through social interaction (6).

Despite strong theoretical and empirical support, the successful implementation of active learning remains complex. The effectiveness of active learning depends not only on the adoption of interactive formats but also on the quality of facilitation, clarity of objectives, and alignment between instructional design and assessment. Poorly structured or inconsistently facilitated active sessions may increase cognitive load, generate frustration, or be perceived as inefficient by learners (7). Moreover, faculty often report using active learning strategies, yet students may experience these sessions as tutor-dominated or minimally interactive, suggesting a potential gap between pedagogical intent and learner experience (2). This discrepancy highlights the importance of implementation fidelity—ensuring that active learning principles are translated into authentic learner engagement.

Empirical classroom-based studies further demonstrate that implementation quality is critical to effectiveness. Andrews et al. (15) found that active learning strategies did not uniformly improve outcomes when facilitation was inconsistent or poorly structured. In addition, Deslauriers et al. (8) showed that students in active learning environments may report lower perceived learning despite objectively higher performance, underscoring the complexity of interpreting satisfaction and perceived engagement as direct indicators of effectiveness.

Faculty development has been identified as a critical factor in improving the effectiveness of active learning. A systematic review by Steinert et al. (9) demonstrated that structured faculty development initiatives can enhance teaching effectiveness and learner outcomes. However, institutions may face structural constraints that limit implementation quality, including large group sizes, limited preparation time, inadequate infrastructure, and insufficient facilitator training. These contextual factors may influence how active learning is experienced in practice.

Cultural and educational traditions may further shape engagement with active learning. In settings where hierarchical or lecture-dominant models have historically predominated, students may require explicit scaffolding to adapt to participatory learning environments. Educational reforms over the past decades have emphasized integration and student-centered learning globally (5), yet the extent to which these reforms translate into meaningful classroom interaction may vary by institutional and sociocultural context.

Cross-cultural research in higher education has demonstrated that participation norms and classroom interaction patterns differ across educational traditions, particularly in contexts characterized by higher power distance and lecture-centered pedagogies. These differences may influence student willingness to engage in open discussion or challenge instructor perspectives, thereby affecting perceptions of active learning effectiveness. Given the limited empirical literature examining active learning implementation in Middle Eastern medical education, situating perception gaps within broader sociocultural frameworks is essential for contextual interpretation.

Within Middle Eastern medical education, empirical studies examining active learning implementation remain comparatively limited. Available regional literature suggests variability in the adoption of active strategies and differences in faculty preparedness across institutions (10). However, few studies have directly compared tutor and student perceptions of how active learning is implemented and experienced within structured tutorial settings. Understanding whether discrepancies exist between intended instructional practices and learner experiences is essential for identifying structural and pedagogical barriers.

At our institution, active teaching and learning strategies are formally embedded within Phase II of the undergraduate medical curriculum through structured tutorial-based sessions designed to promote discussion, collaborative reasoning, and application of knowledge to clinical cases. Informal feedback, however, has suggested variability in engagement and perceived value. Given the strong endorsement of active learning in theory but the variability observed in practice, examining perception gaps within this context may provide important insight into implementation challenges.

This study aimed to examine how active learning is implemented and experienced in tutorial-based sessions in Phase II of an undergraduate medical program by comparing medical students’ and tutors’ perceptions, identifying structural and pedagogical barriers that influence engagement, and generating context-specific recommendations to improve implementation fidelity.

## Materials and methods

### Study design and setting

This was a cross-sectional, mixed-method study conducted in 2024 at the College of Medicine and Health Sciences (CoM&HS), Sultan Qaboos University, Muscat, Oman. The medical degree (MD) program comprises three phases: Phase I (basic sciences); phase II (system-based modules with integrated tutorials), and phase III (clinical clerkships). Weekly small-group tutorials in Phase II are designed to support active learning through case-based discussions. This study focused on tutorials delivered during Phase II modules. In phase II courses, tutorials conducted weekly as clinical case discussions where active teaching and learning should be implemented.

### Participants

Participants included Phase II medical students and faculty members involved in facilitating tutorials. Students in their final semester of Phase II were invited via institutional email, with follow-up reminders to enhance response rates. Faculty were similarly recruited. Participants received an information sheet explaining the study purpose, confidentiality safeguards, and voluntary nature of participation. Completion of the anonymous survey constituted implied informed consent.

Purposive sampling was used for both groups to ensure inclusion of those directly engaged in tutorial activities. For the qualitative component, convenience sampling was used to recruit students from the same cohort for focus groups.

### Data collection

#### Survey instruments

Two structured surveys—one for students and one for tutors—were developed specifically for this study to assess perceptions of active learning implementation. Survey items were generated based on a review of existing medical education literature on active learning and tutorial-based teaching. Domains included frequency of interactive strategies, perceived engagement, satisfaction, alignment with learning objectives, and perceived structural barriers. Both Likert-scale and open-ended items were included.

Content validation was conducted through structured expert review. Four faculty members with experience in medical education and curriculum development independently reviewed the draft instruments for clarity, relevance, and alignment with study objectives. Reviewers were asked to evaluate each item for content relevance and wording clarity and to suggest modifications. Based on this feedback, redundant items were removed, ambiguous wording was revised, and item sequencing was adjusted to improve flow and coherence.

Pilot testing was subsequently conducted with a small group of Phase II students (*n* = 6) and two faculty tutors not participating in the final study. Participants were asked to complete the survey and provide written and verbal feedback regarding clarity, comprehension, and time required for completion. Minor modifications were made to improve phrasing and reduce redundancy.

Given the exploratory nature of the study and the relatively small sample size, formal test–retest reliability and Cronbach’s alpha were not calculated. The surveys were therefore used as context-specific perception tools rather than validated psychometric instruments. The full survey instruments are provided as Supplementary File 1 to ensure transparency and reproducibility.

#### Focused group interviews

Four focus groups were conducted with Phase II students (5–7 participants per group) to explore perceptions of active learning in greater depth. A semi-structured interview guide was developed by the research team based on survey findings and relevant literature on active learning. The guide included open-ended questions exploring engagement, facilitation practices, alignment with objectives, structural barriers, and suggested improvements. The full interview guide is provided as Supplementary File 2.

To minimize potential power dynamics, focus groups were facilitated by trained faculty members who were not directly involved in the design, delivery, or assessment of the tutorial sessions being evaluated. Facilitators had no formal teaching or grading relationship with participating students during the study period. Students were explicitly informed that participation was voluntary, responses would be anonymized, and feedback would not influence academic evaluation.

Sessions were conducted in a neutral classroom setting, audio-recorded with consent, and supplemented by field notes documenting contextual observations and non-verbal cues.

### Data analysis

#### Quantitative analysis

Survey data were analyzed using SPSS v21. Descriptive statistics (means, standard deviations, frequencies, and percentages) were used to summarize participant demographics and responses. Given the exploratory nature of the study, the modest sample size, and the use of non-validated survey instruments, analyses were intentionally limited to descriptive statistics. The study was not designed or powered to detect statistically significant differences between groups, and comparative interpretations are therefore presented cautiously and are supported by qualitative findings rather than formal hypothesis testing.

#### Qualitative analysis

Audio recordings were transcribed verbatim and analyzed using NVivo (version 14, QSR International). Thematic analysis followed Braun and Clarke’s six-phase inductive framework (11).

An initial codebook was developed collaboratively following familiarization with the data. All four focus group transcripts (30–45 min each) were independently double-coded by two researchers to assess coding consistency. Inter-rater reliability was calculated using Cohen’s kappa and demonstrated substantial agreement (*κ* = 0.74). Coding discrepancies were resolved through discussion, and the codebook was refined accordingly. The finalized coding framework was then applied to the full dataset by one researcher, with periodic review by the research team to ensure analytic consistency and coherence.

Field notes documenting contextual observations were integrated into the analysis. Themes were finalized through iterative team discussion to enhance interpretive validity and ensure alignment with participant narratives.

### Ethical considerations

The study received ethical approval from the Medical Research Ethics Committee at the College of Medicine and Health Sciences, Sultan Qaboos University (Approval Number: MREC 3468). All participants were informed about the voluntary nature of participation, confidentiality, and their right to withdraw at any time.

## Results

### Participant demographics

A total of 81 individuals participated, including 52 Phase II medical students and 29 faculty tutors (response rates 33.1 and 23.2%, respectively).

Although recruitment targeted students in the final semester of Phase II (Semester 4), three students from Semester 2 responded to the open survey invitation and were included in descriptive analyses. Because Semester 2 representation was minimal (*n* = 3; 5.8%), no subgroup comparisons were performed, and analyses were conducted on the full student cohort descriptively.

Among students, 55.8% (*n* = 29) were male and 44.2% (*n* = 23) female. The majority (94.2%) were in Semester 4. Tutor demographics showed near-equal gender distribution (48.3% male; 51.7% female). Academic ranks included Assistant Professors (27.6%), Associate Professors (34.5%), and other senior academic or clinical titles (37.9%). Teaching experience ranged from <2 years (17.2%) to >5 years (58.6%) (Table 1).

**Table 1:** Demographic characteristics of student and tutor participants.

Characteristic	Category	Students (*n* = 52)	Tutors (*n* = 29) 2
Gender	Female	23 (44.2%)	14 (48.3%)
	Male	29 (55.8%)	15 (51.7%)
Semester	Semester 2	3 (5.8%)	–
	Semester 4	49 (94.2%)	–
Academic position	Assistant Professor	–	8 (27.6%)
	Associate Professor	–	10 (34.5%)
	Other*	–	11 (37.9%)
Teaching experience	< 2 years	–	5 (17.2%)
	3–5 years	–	7 (24.1%)
	> 5 years	–	17 (58.6%)

*“Other” includes professors, consultants, and senior specialists.

### Student satisfaction and engagement

Overall satisfaction was assessed using the item:“How would you rate your experience with the current tutorial’s teaching?”. A “positive” responses were defined as selecting either *Satisfied* or *Very satisfied*.

Only 5 students (9.6%) reported a positive experience. In contrast, 23 students (44.2%) rated their experience as negative, 23 (44.2%) as neutral, and 1 (1.9%) as very negative (Table 2). These findings indicate that fewer than one in 10 students expressed clear satisfaction with tutorial sessions.

**Table 2:** Student ratings of tutorial experience and engagement.

Measure	Category	Students (*n* = 52)
Overall experience rating	Very negative	1 (1.9%)
	Negative	23 (44.2%)
	Neutral	23 (44.2%)
	Positive	5 (9.6%)
Active engagement frequency	Never	3 (5.8%)
	Rarely (≤10% time)	12 (23.1%)
	Occasionally (≈30%)	13 (25.0%)
	Sometimes (≈50%)	12 (23.1%)
	Frequently (≈70%)	11 (21.2%)
	Usually (≈90%)	1 (1.9%)

Student engagement was assessed using a separate survey item measuring frequency of active participation (e.g., discussion or group work). Only 12 students (23.1%) reported being engaged frequently or usually (≥70% of session time). Most students reported engagement occurring occasionally or sometimes (48.1%), while 15 students (28.9%) reported rare or no engagement (Table 2).

Comfort with participation was similarly modest. Sixteen students (30.7%) reported feeling comfortable or very comfortable contributing during tutorials, whereas 17 (32.7%) reported discomfort or strong discomfort. The remainder (36.5%) reported neutral comfort levels.

### Domain-specific satisfaction

To examine satisfaction across specific tutorial components, responses were analyzed across five domains:

- Clarity of learning objectives
- Teaching methods
- Session organization
- Learning resources
- Tutor support

**Figure 1:**
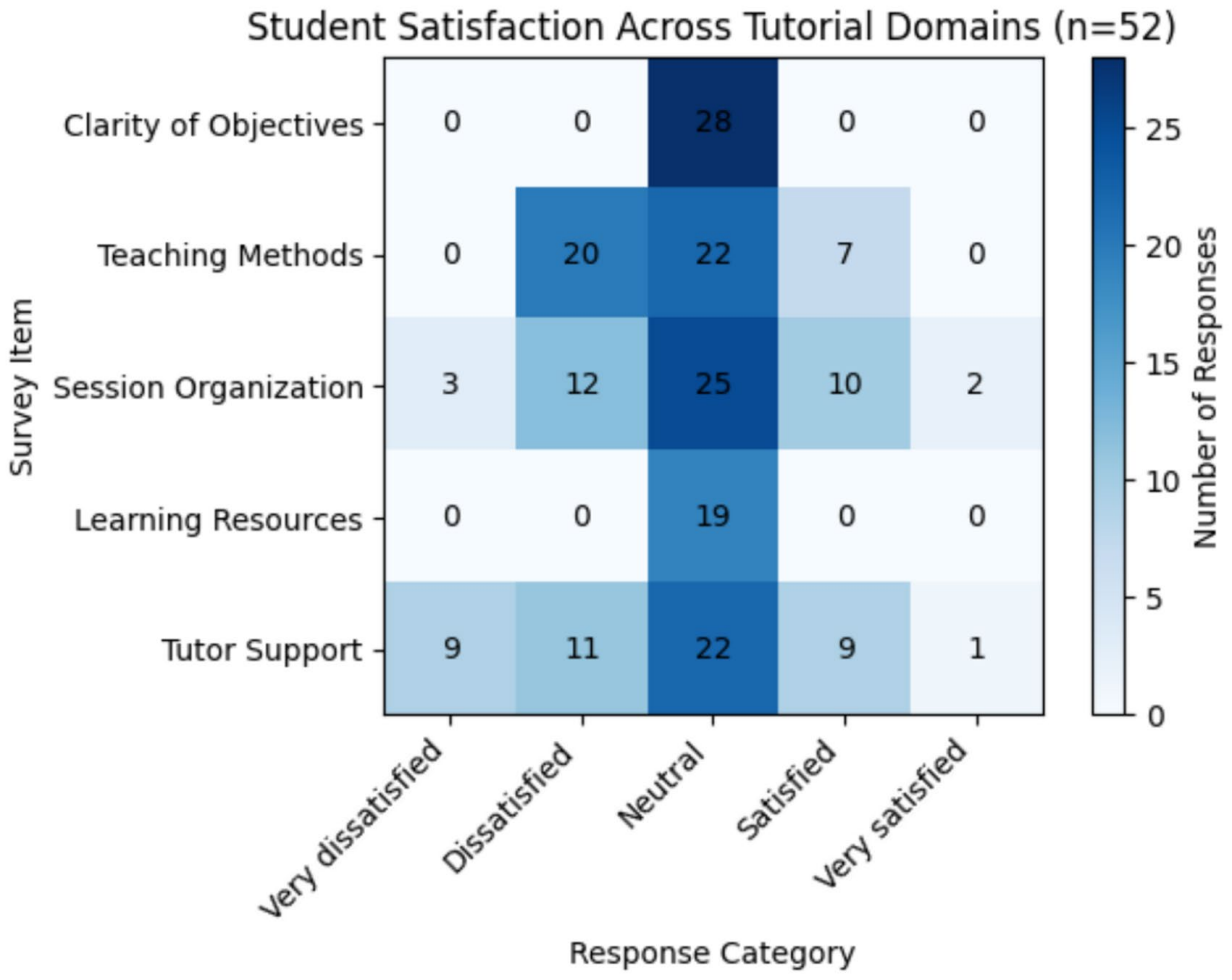
Student satisfaction across tutorial domains (*n* = 52). Heatmap showing distribution of Likert responses (very dissatisfied to very satisfied) across tutorial domains. Numbers represent response counts; darker blue indicates higher frequency.

Figure 1 presents the full distribution of Likert-scale responses across these domains. All question labels have been presented in full for clarity.

Across domains, responses predominantly clustered within neutral or dissatisfied categories. Dissatisfaction was most pronounced for teaching methods and session organization. Satisfaction was relatively higher for tutor support compared with other domains, although positive responses remained limited overall.

Learning environment factors also demonstrated mixed responses. While 46.2% of students reported satisfaction with class size, 17.3% expressed dissatisfaction regarding excessive group size. Satisfaction with physical room setup was reported by 36.5% of students, whereas 40.4% were dissatisfied. Only 19.2% expressed satisfaction with learning resources, and 38.5% reported dissatisfaction.

All data described above correspond directly to Table 2 and Figure 1.

### Tutor practices and barriers to active learning

Tutors reported widespread use of interactive formats, in sharp contrast to student perceptions. All tutors (*n* = 29, 100%) reported using *case-based discussions*, and 27 (93.1%) conducted *small-group discussions*. A majority also used large-group talk (*n* = 18, 62.1%). Other methods were used by smaller subsets: panel discussions 13 (44.8%), simulation exercises 11 (37.9%), and audience-response polling 9 (31.0%). These usage rates are summarized in Table 3.

**Table 3:** Teaching methods used by faculty tutors during tutorials (*n* = 29).

Teaching method	Tutors (*n* = 29)
Case-based discussion	29 (100.0%)
Small-group discussion	27 (93.1%)
Large-group discussion	18 (62.1%)
Panel discussion	13 (44.8%)
Simulation exercises	11 (37.9%)
Audience response polling	9 (31.0%)

Despite this, tutors acknowledged practical barriers. When asked about challenges to active learning, 20 tutors (69.0%) cited the *large number of students*, 18 (62.1%) noted *insufficient training*, and 14 (48.3%) cited *lack of preparation time*. Many tutors mentioned inadequate classroom infrastructure and curricular time constraints. Notably, virtually all tutors (100%) expressed interest in additional training or workshops on active learning methods.

### Qualitative findings

Thematic analysis of student focus groups identified three primary themes.

1. Tutor-dominated sessions with limited interactivity

Students frequently described tutorials as resembling mini-lectures rather than interactive sessions. Several participants reported limited opportunities to contribute to discussion. One student stated, “It felt like a mini-lecture every time—we were mostly listening to the tutor instead of discussing.” Another commented, “I hardly got a chance to talk or solve problems myself.”

2 Misalignment with learning objectives

Participants reported that tutorial content was sometimes unclear or repetitive. Students noted that objectives were not always explicitly addressed during sessions. One student remarked, “Sometimes the case objectives are unclear or repeat lecture points.” Others described tutorials as revisiting previously covered material without adding new understanding.

3 Structural and environmental constraints

Group size and physical environment were frequently identified as barriers to participation. Students described overcrowded rooms and limited space for discussion. One participant stated, “With 30 people in the room, it’s difficult for everyone to participate.” Concerns regarding classroom layout and audiovisual limitations were also raised.

In tutor open-ended survey responses, an additional theme emerged:

4 Need for facilitator preparation and training

Tutors acknowledged challenges related to limited training and preparation time. Several reported defaulting to lecture-style delivery due to lack of structured guidance on facilitating interactive tutorials.

## Discussion

This mixed-methods study identified a potential perception gap between tutors’ perceptions of active learning implementation and students’ lived experiences within tutorial-based sessions. Although tutors reported frequent use of interactive strategies, students largely experienced tutorials as passive, tutor-dominated, and minimally engaging. These findings suggest that the structural adoption of active learning formats does not necessarily translate into meaningful learner participation. Integration of quantitative and qualitative findings strengthens the interpretation of these results. While survey data suggested low student satisfaction and engagement, qualitative findings provided explanatory depth, revealing that tutor-dominated delivery, unclear objectives, and structural constraints contributed to these perceptions. Rather than representing independent findings, the qualitative data contextualize and help explain the patterns observed in the quantitative results, supporting a convergent interpretation of limited perceived interactivity despite reported use of active strategies.

Active learning is consistently associated with improved academic performance and engagement when implemented effectively (1, 2). Structured approaches such as problem-based learning emphasize clearly defined facilitation roles, alignment between objectives and discussion processes, and active student participation (3). Faculty development has also been shown to strengthen teaching effectiveness and support meaningful learner engagement (9). However, students’ perceptions of learning do not always align with instructional intentions or objective learning outcomes. Deslauriers et al. (8) demonstrated that learners in active classrooms may report lower perceived learning despite superior performance. Similarly, Testa et al. (12) found that faculty may overestimate the use of active learning strategies compared with student recognition of those methods. The perception gap observed in the present study aligns with these findings, although the very low proportion of students reporting positive satisfaction (<10%) is notable.

Several contextual factors likely contributed. Group size emerged as a major barrier, limiting opportunities for distributed participation despite the emphasis on small-group engagement in the literature (2). Tutors also identified limited preparation time and insufficient facilitation training. Faculty development is strongly associated with improved teaching effectiveness (9); without it, tutorials may revert to lecture-style delivery despite interactive intentions. In addition, students described misalignment between objectives and session activities, indicating weaknesses in instructional coherence. Effective active learning requires alignment between case design, objectives, and facilitation processes (3); when such alignment is lacking, sessions may be perceived as repetitive rather than additive.

These findings suggest variability in implementation fidelity, although this was not directly measured. Although tutorials were formally designated as active learning sessions, essential elements such as distributed participation, structured questioning, and student-led reasoning were inconsistently enacted. Educational research cautions that nominal adoption of active formats does not ensure cognitive engagement (2).

Cognitive Load Theory offers one explanatory perspective. Unclear objectives, repetition, and overcrowding likely increased extraneous cognitive load, reducing capacity for meaningful processing (7). From a motivational standpoint, Self-Determination Theory posits that engagement depends on support for autonomy and competence (13). Limited opportunities to participate and discomfort contributing during tutorials may indicate unmet motivational needs, further explaining low engagement despite tutors’ reported efforts.

Although conducted within a single institution, the present findings contribute to the growing international literature examining perception gaps in active learning implementation. Empirical studies have documented discrepancies between instructor perceptions of active teaching practices and student-reported classroom engagement. For example, Testa et al. (12) demonstrated that faculty frequently overestimate their use of active learning strategies compared with student recognition of those methods. Similarly, Deslauriers et al. (8) showed that students’ perceptions of learning may not align with objective performance outcomes in active learning environments, highlighting the complexity of interpreting satisfaction as a proxy for effectiveness. While large-scale meta-analyses have consistently demonstrated the benefits of active learning on performance (1), reported student satisfaction in many Western contexts tends to be moderate to high. The markedly low satisfaction observed in the present study suggests that contextual and structural factors may contribute to amplifying perception gaps within specific institutional environments. By directly comparing tutor and student perceptions within the same instructional setting, this study extends prior research by quantifying the magnitude of this discrepancy in an underreported Middle Eastern medical education context (10) and identifying structural contributors to its persistence. Similar perception patterns have been reported in other Middle Eastern healthcare education contexts. For example, Kalu et al. (14) found that nursing students in Qatar valued interactive strategies but reported variability in facilitation quality and structural barriers to participation. Although conducted in a different discipline, these findings reinforce the importance of contextual and cultural factors in shaping active learning experiences across the region.

### Limitations

This study was conducted at a single institution, which may limit generalizability. The survey instruments were study-specific and not formally validated; although content validity was supported through expert review and pilot testing, the absence of construct validation and reliability testing introduces potential measurement error. Accordingly, findings should be interpreted as reflecting perceived patterns rather than precise estimates, and differences between students and tutors may partly reflect variation in item interpretation. The quantitative analysis was limited to descriptive statistics, consistent with the exploratory design and sample size, and does not support inferential comparisons. Qualitative findings, while rigorously analyzed, remain interpretive. In addition, tutor responses may have been influenced by social desirability bias. These limitations highlight the need for future studies using validated instruments and broader samples.

## Conclusion

This study demonstrates that discrepancies between intended and experienced active learning persist despite widespread endorsement of active teaching strategies. To enhance implementation fidelity in tutorial settings, the following actions are recommended:

- Structured faculty development in facilitation skills
- Reduction of tutorial group size to promote participation
- Explicit alignment between tutorial cases and learning objectives
- Improved physical learning environments
- Ongoing monitoring of tutorial quality and implementation consistency.

Addressing these structural and pedagogical factors may transform tutorials from nominally active sessions into genuinely engaging learning experiences.

## Data Availability

The raw data supporting the conclusions of this article will be made available by the authors, without undue reservation.

## References

[1] Freeman S Eddy SL McDonough M Smith MK Okoroafor N Jordt H . Active learning increases student performance in science, engineering, and mathematics. Proc Natl Acad Sci. (2014) 111:8410–5. doi: 10.1073/pnas.1319030111, 24821756 PMC4060654

[2] Prince M. Does active learning work? A review of the research. J Eng Educ. (2004) 93:223–31. doi: 10.1002/j.2168-9830.2004.tb00809.x

[3] Schmidt HG Rotgans JI Yew EHJ. The process of problem-based learning: what works and why. Med Educ. (2011) 45:792–806. doi: 10.1111/j.1365-2923.2011.04035.x, 21752076

[4] Harden RM. The integration ladder: a tool for curriculum planning and evaluation. Med Educ. (2000) 34:551–7. doi: 10.1046/j.1365-2923.2000.00697.x 10886638

[5] Irby DM Cooke M O’Brien BC. Calls for reform of medical education by the Carnegie Foundation: 1910 and 2010. Acad Med. (2010) 85:220–7. doi: 10.1097/ACM.0b013e3181c88449 20107346

[6] Vygotsky LS. Mind in Society: The Development of Higher Psychological Processes. Cambridge: Harvard University Press (1978).

[7] Young JQ Sewell JL. Applying cognitive load theory to medical education: AMEE guide no. 86. Med Teach. (2014) 36:371–84. doi: 10.3109/0142159X.2014.889290 24593808

[8] Deslauriers L McCarty LS Miller K Callaghan K Kestin G. Measuring actual learning versus feeling of learning in response to being actively engaged in the classroom. Proc Natl Acad Sci. (2019) 116:19251–7. doi: 10.1073/pnas.1821936116, 31484770 PMC6765278

[9] Steinert Y Mann K Centeno A Dolmans D Spencer J Gelula M . A systematic review of faculty development initiatives designed to improve teaching effectiveness in medical education: BEME guide no. 8. Med Teach. (2006) 28:497–526. doi: 10.1080/01421590600902976, 17074699

[10] AlRuthia Y Alhawas S Alodaibi F Almutairi L Algasem R Alrabiah HK . The use of active learning strategies in healthcare colleges in the Middle East. BMC Med Educ. (2019) 19:143. doi: 10.1186/s12909-019-1580-4, 31088430 PMC6518770

[11] Braun V Clarke V. Using thematic analysis in psychology. Qual Res Psychol. (2006) 3:77–101. doi: 10.1191/1478088706qp063oa

[12] Testa GMG Souza MBO Paes ÂT Magdalon J. Students outperform faculty in recognizing the use of active learning methods. Adv Physiol Educ. (2025) 49:356–65. doi: 10.1152/advan.00132.2024, 39925088

[13] Ryan RM Deci EL. Self-determination theory and the facilitation of intrinsic motivation, social development, and well-being. Am Psychol. (2000) 55:68–78. doi: 10.1037/0003-066X.55.1.68, 11392867

[14] Kalu F Wolsey C Enghiad P. Undergraduate nursing students' perceptions of active learning strategies: a focus group study. Nurse Educ Today. (2023) 131:105986. doi: 10.1016/j.nedt.2023.105986, 37857101

[15] Andrews TM Leonard MJ Colgrove CA Kalinowski ST. Active learning not associated with student learning in a random sample of college biology courses. CBE Life Sci Educ. (2011) 10:394–405. doi: 10.1187/cbe.11-07-0061, 22135373 PMC3228657

