# Supplementary material for: Perception and implementation gaps in active teaching and learning: a mixed-methods study of students and tutors in a medical degree program
Source: Front Med (Lausanne). 2026 May 21;13:1796205. doi: 10.3389/fmed.2026.1796205 (PMC13237709; doi:10.3389/fmed.2026.1796205)
Supplement: Supplementary file 1 [file Supplementary_file_1.pdf]

Students' survey: Assessment of Teaching strategies for Phase II **tutorials** in Integrated Modules and System Based Courses to improve learning outcomes

Thank you for participating in
this survey to assess students' satisfaction with tutorials as part of Phase II integrated modules and system-based courses. Your contributions are essential in allowing us to complete this study and improve the students' and tutors' experience. The survey will take 5-10 minutes to complete, and all responses will be kept confidential and de-identified. Please feel free to provide additional comments on the final page of the survey. All feedback will be reviewed and appreciated. Thank you again for contributing to this study; your input is extremely valuable.

Definitions:

Tutors: Teachers who conduct tutorials in Phase II/integrated modules/system-based courses

Tutorials: Phase II integrated modules/system-based courses tutorials

AL: Active learning.

Contact info: If you have any questions, please don't hesitate to send an email to Ibrahimn@squ.edu.om

* Indicates required question

Students basic information

1. Gender *

Mark only one oval.

- Male
- Female

2. 2. What Phase II semester are you in currently? *

Mark only one oval.

- Semester 1
- Semester 2
- Semester 3
- Semester 4

Students' stratification/Perceptions during tutorials teachings

3. 3. How well do you feel the tutorial sessions meet the stated objectives for each tutorial? *

Mark only one oval.

- Very good
- Good
- Acceptable
- Poor
- Very poor

4. 4. How effective do you think the tutorial teaching methods are in helping you learn and retain information? *

Mark only one oval.

- Very effective
- Effective
- Neutral
- Ineffective
- Very ineffective

5. 5. How would you rate the overall effectiveness of tutorial teaching in helping you succeed in your course? *

Mark only one oval.

- Very effective
- Effective
- Neutral
- Ineffective
- Very ineffective

6. 6. How often are you actively engaged during tutorials? (e.g., through discussions or group work) *

Mark only one oval.

- Every time
- Usually (i.e about 90% of the time)
- Frequently (i.e about 70% of the time)
- Sometimes (i.e about 50% of the time)
- Occasionally (i.e about 30% of the time)
- Rarely (i.e less than 10% of the time)
- Never

7. 7. How comfortable do you feel participating in the tutorial discussion? *

Mark only one oval.

- Very comfortable
- Comfortable
- Neutral
- Uncomfortable
- Very uncomfortable

8. 8. How satisfied are you with the availability of learning resources for tutorial sessions? (e.g. reading references, handouts, online materials). *

Mark only one oval.

- Very satisfied
- Satisfied
- Neutral
- Dissatisfied
- Very dissatisfied

9. 9. How satisfied are you with tutors' support and guidance during tutorial sessions? *

Mark only one oval.

- Very satisfied
- Satisfied
- Neutral
- Dissatisfied
- Very dissatisfied

10. 10. How satisfied are you with the organization of the tutorial sessions? *

Mark only one oval.

- Very satisfied
- Satisfied
- Neutral
- Dissatisfied
- Very dissatisfied

11. 11. Are you satisfied with the number of students per tutorial session? *

Mark only one oval.

- Very satisfied
- Satisfied
- Neutral
- Dissatisfied
- Very dissatisfied

12. 12. Are you satisfied with the tutorial room setup? (e.g. layout, size, lighting, audiovisual tools). *

Mark only one oval.

- Very satisfied
- Satisfied
- Neutral
- Dissatisfied
- Very dissatisfied

13. 13. How would you rate your experience with the current tutorial's teaching? *

Mark only one oval.

- Very positive
- Positive
- Neutral
- Negative
- Very negative

14. 14. Which of the following phase II courses, in your opinion, provide the best tutorial experience? (Choose all that apply) *

Check all that apply.

- [ ] Endocrine System
- [ ] Human Nervous System
- [ ] Locomotor System
- [ ] Uro-reproductive System
- [ ] Alimentary System
- [ ] Cardiovascular System
- [ ] Respiratory System
- [ ] Hematopoietic and Immune Systems
- [ ] Integrated Module I
- [ ] Integrated Module II
- [ ] Integrated Module III

15. 15. What would increase your **attendance** in the tutorial sessions? (choose all that apply) *

Check all that apply.

- [ ] Student lead presentations
- [ ] Pre tutorials quizzes
- [ ] Team-based learning or peer-to-peer learning
- [ ] Incentives with marks given to attendance
- [ ] Modifying current teaching styles to be more interactive and engaging

16. 16. What would increase your **engagement** in the tutorial sessions? (choose all that apply) *

Check all that apply.

- [ ] Student lead presentations
- [ ] Pre tutorials quizzes
- [ ] Team-based learning or peer-to-peer learning
- [ ] Incentives with marks given to participation
- [ ] Modifying current teaching styles to be more interactive and engaging

17. 17. What do you like most about the tutorials teaching? *

18. 18. What aspects of tutorial teaching need improvement? *

This content is neither created nor endorsed by Google.

Google Forms

Tutors Survey: Assessment of Teaching strategies for Phase II **tutorials** in Integrated Modules and System Based Courses

Thank you for participating in this survey to assess tutors' teaching methodology for tutorials as part of Phase II integrated modules and system-based courses. Your contributions are essential in allowing us to complete this study and improve the students' and tutors' experience. The survey will take 5-10 minutes to complete, and all responses will be kept confidential and de-identified. Please feel free to provide additional comments on the final page of the survey. All feedback will be reviewed and appreciated. Thank you again for contributing to this study; your input is extremely valuable.

Definitions:

Tutors: Teachers who conduct tutorials in Phase II/integrated modules/system-based courses

Tutorials: Phase II integrated modules/system-based course tutorials

AL: Active Learning

Contact info: if you have any questions, please don't hesitate to send an email to Ibrahimn@squ.edu.om

* Indicates required question

Tutors basic information

1. Please select one of the options in each of the categories below related to your age/gender/title:

1. Age *

Mark only one oval.

- 25-34
- 35-44
- 45-54
- 55-64
- 65+

2. Gender *

Mark only one oval.

- Male
- Female

3. Title/Academic position *

Mark only one oval.

- Clinical lecturer
- Assistant prof
- Associate prof
- Professor
- Senior Consultant
- Consultant
- Senior Specialist

4. 2. What level of medical education certificates/training do you have? (choose all that apply) *

Check all that apply.

- ☐ Post MD masters in education /Ph.D. in education
- ☐ Masters in education /Ph.D. in education (for none MD holders)
- ☐ Attended workshops on teaching methods in the last 3 years
- ☐ I have no formal training but learned strategies from experience with colleagues
- ☐ Other: __________

5. 3. How long have you been teaching undergraduate tutorials for Phase II courses? *

Mark only one oval.

- ◯ <2 years
- ◯ 3-5 years
- ◯ >5 years

6. 4. Do you hold an MD degree? *

Mark only one oval.

- ◯ Yes
- ◯ No

7. 5. Do you hold a basic science PhD degree? *

Mark only one oval.

- ◯ Yes
- ◯ No

8. 6. What is your administrative affiliation? *

Mark only one oval.

- SQUH (UMC)
- College of Medicine and Health Science
- Other: ____

Current teaching environment/methods

9. 7. I use and/or allow the students to use the following methods for teaching the tutorials *

Mark only one oval per row.

	Yes	No	I don't know/I am unsure
**Case-based discussion**	⬭	⬭	⬭
**Panel discussion**	⬭	⬭	⬭
**Simulation exercises**	⬭	⬭	⬭
**Audience Response Polling**	⬭	⬭	⬭
**Small group discussion**	⬭	⬭	⬭
**Large group discussion**	⬭	⬭	⬭
**Self-reflection exercises**	⬭	⬭	⬭
**Think-Pair-Share**	⬭	⬭	⬭
**Flipped classroom**	⬭	⬭	⬭
**Peer observation and feedback**	⬭	⬭	⬭
**Turn and Talk**	⬭	⬭	⬭

**Pause procedures during lecture**			
**Bulleted breaks during the lecture**			
**One-minute paper**			
**Other method not mentioned above**			

10. 8. I do use the following teaching tools/aids with my teaching *during tutorials* (choose all that apply): *

Check all that apply.

- [ ] PowerPoint slides
- [ ] Whiteboard/marker
- [ ] Verbal discussion
- [ ] Other: ____________

11. 9. What is the main use of PowerPoint slides if you use PowerPoint slides? *

Mark only one oval.

- Providing visual aids such as images, graphs, and charts
- I put the cases' questions and answers in PowerPoint slides, and I use this to guide the audience through the content being discussed
- I use PowerPoint to provide sharable content to students at the end of the tutorial session for their exam study/review
- I use PowerPoint slides to help me maintain audience engagement and improve retention of information by breaking down complex topics into more digestible segments
- I don't use PowerPoint slides

12. 10. Are you satisfied with your teaching strategies for tutorials? *

Mark only one oval.

- Very satisfied
- Satisfied
- Neutral
- Dissatisfied
- Very dissatisfied

13. 11. Would you be interested to learn about other teaching strategies in the future? * (e.g. one day workshop)

Mark only one oval.

- Yes
- No
- Maybe

14. 12. Describe your teaching strategy on tutorials briefly *

Attitudes/Knowgele/skills in AL

15. 13. Are you familiar with the concept of active learning during tutorials? *

Mark only one oval.

- Yes
- No
- I have some partial knowledge/vague knowledge

16. 14. What is your understanding of AL? Please select from the following agreement scale for each of the statements below regarding active learning. *

Mark only one oval per row.

	Strongly agree	agree	Neither agree or disagree	disagree	Strongly disagree
**AL combines engagement and observation with reflection**	◯	◯	◯	◯	◯
**The learner in AL is engaged so that both knowledge gained and recall are increased**	◯	◯	◯	◯	◯
**Both the instructor and the learners work cooperatively in AL.**	◯	◯	◯	◯	◯
**With AL, instructors are more concerned with eliciting reflective thoughts that apply knowledge to practice than merely conveying facts.**	◯	◯	◯	◯	◯
AL changes the teacher-					

AL changes the teacher-learner relationship to a learner-learner relationship.					
Lectures (passive learning) have difficulty adapting to AL methods					

17. 15. What challenges do you currently foresee in implementing active learning in Phase II tutorials? (Select all that apply) *

Check all that apply.

- [ ] Lack of time to prepare for active learning activities
- [ ] Insufficient training or resources
- [ ] Resistance from students
- [ ] Inadequate classroom infrastructure
- [ ] Institutional policies or curriculum constraints
- [ ] Large number of students in the tutorials (>25 students).
- [ ] Other: ______________________

18. 16. Please rate each resource's usefulness for increasing active learning in your tutorials. Use the scale from 1 (Not at all useful) to 5 (Extremely useful). *

Mark only one oval per row.

	1	2	3	4	5
**Peer mentoring/review**	◯	◯	◯	◯	◯
**Active self-learning: e.g videos, leaflets**	◯	◯	◯	◯	◯
**Assistance from the medical education department**	◯	◯	◯	◯	◯
**Recognition by the university/college for AL teaching**	◯	◯	◯	◯	◯
**University/college-wide training workshops on AL**	◯	◯	◯	◯	◯
**Publications on best practices on AL**	◯	◯	◯	◯	◯

This content is neither created nor endorsed by Google.

Google Forms
