# Supplementary material for: Perception and implementation gaps in active teaching and learning: a mixed-methods study of students and tutors in a medical degree program
Source: Front Med (Lausanne). 2026 May 21;13:1796205. doi: 10.3389/fmed.2026.1796205 (PMC13237709; doi:10.3389/fmed.2026.1796205)
Supplement: Supplementary file 2 [file Supplementary_file_2.pdf]

Title: Focus Group Guide – Student Perceptions of Active Learning in Tutorial Sessions

Purpose:
To explore student experiences, perceptions, and barriers related to active learning implementation within Phase II tutorial sessions.

Opening Script (Read to Participants):
Thank you for participating. The purpose of this discussion is to understand your experiences with tutorial-based learning. There are no right or wrong answers. We are interested in your honest perspectives. Your responses will remain confidential, and no names will be used in reporting. Participation is voluntary, and you may choose not to answer any question.

Core Questions:

1. How would you describe your overall experience with tutorial sessions in Phase II?
2. To what extent do you feel tutorials promote active participation?
3. Can you describe what typically happens during a tutorial session?
4. What strategies do tutors use to encourage discussion or engagement?
5. Do you feel you have sufficient opportunities to contribute? Why or why not?
6. How well do tutorial sessions align with the stated learning objectives?
7. What factors help facilitate active learning during tutorials?
8. What barriers limit engagement or participation?
9. How does group size affect your learning experience?
10. What changes would improve tutorial effectiveness?
11. Do you feel prepared for active participation? Why or why not?
12. Is there anything else you would like to add about tutorials?

Probing Prompts (Used as Needed):

- Can you give an example?
- How did that make you feel?
- What would have worked better?
- Does anyone have a different experience?

Duration: 30–45 minutes
Facilitator role: Neutral moderation, encouraging balanced participation.
